# Skill Proficiency is Predicted by Intubation Frequency of Emergency Medicine Attending Physicians

**DOI:** 10.5811/westjem.2019.6.42946

**Published:** 2019-07-02

**Authors:** Brian Gillett, David Saloum, Amish Aghera, John P. Marshall

**Affiliations:** Maimonides Medical Center, Department of Emergency Medicine, Brooklyn, New York

## Abstract

**Introduction:**

Airway management is a fundamental skill of emergency medicine (EM) practice, and suboptimal management leads to poor outcomes. Endotracheal intubation (ETI) is a procedure that is specifically taught in residency, but little is known how best to maintain proficiency in this skill throughout the practitioner’s career. The goal of this study was to identify how the frequency of intubation correlated with measured performance.

**Methods:**

We assessed 44 emergency physicians for proficiency at ETI by direct laryngoscopy on a simulator. The electronic health record was then queried to obtain their average number of annual ETIs and the time since their last ETI, supervised and individually performed, over a two-year period. We evaluated the strength of correlation between these factors and assessment scores, and then conducted a receiver operator characteristic (ROC) curve analysis to identify factors that predicted proficient performance.

**Results:**

The mean score was 81% (95% confidence interval, 76% – 86%). Scores correlated well with the mean number of ETIs performed annually and with the mean number supervised annually (r = 0.6, p = 0.001 for both). ROC curve analysis identified that physicians would obtain a proficient score if they had performed an average of at least three ETIs annually (sensitivity = 90%, specificity = 64%, AUC = 0.87, p = 0.001) or supervised an average of at least five ETIs annually (sensitivity = 90%, specificity = 59%, AUC = 0.81, p = 0.006) over the previous two years.

**Conclusion:**

Performing at least three or supervising at least five ETIs annually, averaged over a two-year period, predicted proficient performance on a simulation-based skills assessment. We advocate for proactive maintenance and enhancement of skills, particularly for those who infrequently perform this procedure.

## INTRODUCTION

Endotracheal intubation (ETI) is a high-stakes, life-saving, procedural skill. However, little is understood regarding maintenance of proficiency for this procedure. Although airway skills are known to decline without continuous practice,^1^,^2^ factors influencing the maintenance of proficiency for this procedure are poorly understood. Patients requiring ETI for impending respiratory failure are at high risk of death or permanent cognitive impairment when the procedure is improperly performed. Prior studies demonstrate that patients undergoing multiple attempts at intubation (three or more) have significantly higher adverse event rates as compared with patients undergoing fewer attempts.^3^,^4^

The Accreditation Council of Graduate Medical Education (ACGME) mandates that resident physicians, prior to graduation, perform a specific number of intubations, which varies across specialties, in order to achieve proficiency for this skill.^5^ However, once acquired, the best way to maintain this skill is unclear,^6^,^7^ and evidence is lacking regarding the minimum experience needed to maintain proficiency. Pusic et al. suggested that there is a rate for both gaining and losing skills, and that deliberate practice was the method of choice for avoiding losses.^8^

The Agency for Healthcare Research and Quality, The Joint Commission, and the American Board of Medical Specialties (ABMS) have increased efforts on quality improvement initiatives that facilitate the maintenance of proficiency and adherence to evidence-based standards. In 2004, the American Board of Emergency Medicine (ABEM) implemented a maintenance of certification (MOC) program to improve the quality of care delivered by emergency physicians.^9^ It consists of the four components proposed by ABMS: 1) lifelong learning and self-assessment; 2) improvement in medical practice; 3) the ConCert examination (assessment of knowledge, judgment and skills); and 4) professionalism and professional standing. Despite these efforts, there is no evidence-based guideline for maintaining proficiency in procedural skills such as ETI.

The purpose of this study was to identify factors relating to intubation frequency that correlate with proficiency for ETI.

## METHODS

### Study Design

This was a cross-sectional study to determine factors related to intubation frequency that correlated with assessed skill of ETI via direct laryngoscopy (DL) on an airway simulator. We performed a subsequent analysis on factors with good correlation to identify intubation frequencies that could predict assessment scores below a defined proficiency level. The study was classified as “exempt” by the local institutional review board.

### Study Setting and Sample

The study was conducted at a private urban hospital in the Northeast with an annual emergency department census of 115,000 patients, and an associated emergency medicine (EM) residency program. Subjects assessed for ETI proficiency included a convenience sample of all employed full-time attending physicians over a three-month time period. All participants were board certified or board eligible in EM, pediatric EM, or both. Participation was mandated as part of a departmental skills advancement initiative conducted between November 2011 and January 2012.

### Measurements

The primary outcome measures were the strength of correlation between DL ETI assessment scores and the following: 1) the time since last performing an intubation; 2) the time since last supervising an intubation; 3) the mean number of intubations performed annually; and 4) the mean number of intubations supervised annually. The secondary outcome measure was the identification of intubation frequencies that predict a physician score below the established proficiency score on the airway assessment. We performed a post hoc analysis to measure the strength of correlation with years of experience and ETI assessment score.

**Population Health Research Capsule**

What do we already know about this issue?

Intubation skill is associated with frequency of performance and deliberate practice, not years of experience. Formal skill assessment after residency is uncommon.

What was the research question?

How does the frequency of endotracheal intubation correlate with measured performance in attending physicians?

What was the major finding of the study?

Performing at least three or supervising at least five intubations annually predicted proficient skill.

How does this improve population health?

Evidence-based guidelines regarding intubation frequency help inform the need for proactive training to maintain proficiency in this critical skill.

#### ETI Assessment - Score Calculation

Each attending physician was individually administered a skills assessment of ETI by DL on a TruCorp Airsim Advance mannequin, which was a model replicating the airway from DICOM library images of an actual patient’s computed tomography. We assessed physicians for successful completion of 11 checklist items (Table 1), as well as their overall psychomotor adeptness using a rating scale of 0–10, with 0 representing significant “struggle” and 10 representing “no struggle.” The construct of “struggle” was defined by characteristics such as coordination, grace, dexterity, and timing. Of note, time itself was not discretely measured as we aimed for the assessment to reward quality over speed. At least two of three pre-trained raters were present during each evaluation, and performance scores were recorded by consensus on a standardized evaluation form. Successful performance for each checklist item was grounded in common best practices. For example, the item “inserts tube to correct depth” would be considered acceptable if a 7.5 tube was placed at a level anywhere from 21–24 cm at the lips. Regarding tube and blade size, a variety of common options available in the clinical space were provided, and any reasonable choice appropriate to the size of the airway simulator was deemed acceptable. The binary evaluation for procedural step completion and the overall psychomotor adeptness scale were similarly weighted such that the total assessment had a maximal potential score of 21 points. The total score was ultimately represented as a percentage of the total possible points (ie, 18/21 would be 86%).

#### ETI Assessment - Instrument Validity

There is no well-established, validated tool for measuring ETI skill via the DL approach for experienced providers who served as our population group. Furthermore, few previously published tools provide validity evidence in accordance with its contemporary conceptualization embodied by the current Standards of Educational and Psychological Testing.^10^ Thus, the authors chose to develop a novel assessment tool. Content validity of the tool is supported in that checklist items were crafted after triangulation of multiple sources for best practices in EM and anesthesiology textbooks and discussion with experienced emergency physicians.^11^,^12^

Due to the inherent limitations in an assessment rooted purely in checklist items,^13^ we also used a psychomotor scale to evaluate other characteristics of procedural skill such as coordination, grace, dexterity, and timing. Checklists alone have been criticized for rewarding thoroughness rather than competence,^14^ and do not differentiate the novice who performs all steps (poorly) from the expert. They do add an objective component to the evaluation that allows assessors a standardized report of critical actions. Global rating scales may be more appropriate for assessment on performance-based evaluations,^15^ and have been shown to have good psychometric characteristics when used in conjunction with a checklist.^16^,^17^

Response process was supported in that all three raters (authors BG, DS, and AA) were involved in developing the instrument and had come to consensus on how to employ the tool a priori. Raters also familiarized themselves with equipment and testing conditions in advance of assessments, and they deliberated upon ratings for each step, with disagreements discussed in real time until there was consensus. As the scores were shared with the department chair as part of Ongoing Professional Practice Evaluations, care was taken to ensure that scores were an accurate representation of performance on the simulator. This was made explicitly transparent to participants, thus providing an impetus to make their best attempt at intubating as if it were a real patient. Further consequences validity evidence was provided in that low assessment scores resulted in protected time to attend an airway skills refresher course at the expense of the department, additional mannequin training in the simulation center, and repeat assessment (with improved results). Validity evidence based on internal structure of the tool was supported by demonstration of good internal consistency between checklist items and psychomotor adeptness (Cronbach’s α = 0.8).

#### ETI Assessment - Proficiency Cut Score

We used a borderline methods approach to determine a cut score for proficient skill performance.^18^ The construct of proficient performance was defined as a physician demonstrating requisite skill such that he or she is likely to successfully intubate patients via DL in the emergent setting, consistent with the definition of proficiency as provided by Dreyfus.^19^ After participants completed their assessment, each rater independently identified attendings whose performance was not clearly proficient or clearly not proficient, ie, on the borderline. We used the median score from this cohort as the cut off for proficient skill performance.

### Data Analysis

We presented assessment scores and intubation frequencies with descriptive statistics and 95% confidence intervals (CI). Intubation frequencies were obtained by querying the electronic health record over the previous two years, concluding on the date of each physician’s assessment. Factors relating to intubation frequency were 1) the time interval between a physician’s assessment and their last performance of an ETI; 2) the time interval between a physician’s assessment and their last supervision of an ETI; 3) the total number of ETIs performed; and 4) the total number of ETIs supervised. We performed a post hoc analysis to measure the strength of correlation with years of experience and ETI assessment score.

The strength of correlation between assessment scores and each of these factors was calculated using Pearson’s correlation coefficient. Factors that demonstrated good correlation with assessment scores (r ≥ 0.6) were plotted on a receiver operating characteristic (ROC) curve to identify specific values that would predict ETI assessments below the proficiency cut score. We evaluated internal consistency of the assessment tool with Cronbach’s alpha for its two overarching aspects, psychomotor adeptness and completion of procedural steps. Data was analyzed with SPSS version 20 (IBM, Armonk, New York).

## RESULTS

We assessed all full-time employed EM attending physicians (n = 44, 33 general EM trained and pediatric EM (PEM) trained). From this initial cohort, 12 were excluded as they were not present for the entirety of the two-year, look-back period, leaving 24 EM-trained and 8 PEM-trained physicians (n = 32). The mean years of professional practice for the physician group, defined as years practiced since graduating from residency, was 10.3 years (95% CI, 7.4–13.3) (Table 2).

General emergency physicians on staff during the look-back period performed an average of 4.2 intubations per year (95% CI, 2.8–5.6) and supervised an average of 5.3 per year (95% CI, 4.4–6.2). PEM physicians on staff during the two-year, look-back period performed an average of 0.2 intubations per year (95% CI, 0–0.4) and supervised an average of 0.3 per year (95% CI, 0.1–0.6). There was significant heterogeneity between physicians regarding the number of days elapsed between taking the assessment and last performing an intubation (mean = 405, median = 74, standard deviation = 687) or last supervising an intubation (mean = 83, median = 35, standard deviation = 144). A summary of EM and PEM assessment scores is provided in Table 3.

We identified 14 participants as borderline performers (10 EM and 4 PEM) relating to the construct of clearly evident proficient performance. The median assessment score for the borderline group was 79% (lower quartile = 75%; upper quartile = 86%,; interquartile range = 11%).

Scores correlated well with the average number of intubations performed per year (r = 0.6, p < 0.001) and with the average number of intubations supervised per year (r = 0.6, p = 0.001). Scores did not correlate as well with the time passed since last supervising or performing an intubation, or with years of experience (r = −0.5, p = 0.002; r = −0.3, p = 0.07; and r = −0.4, p = 0.004; respectively).

**Figure 1:**
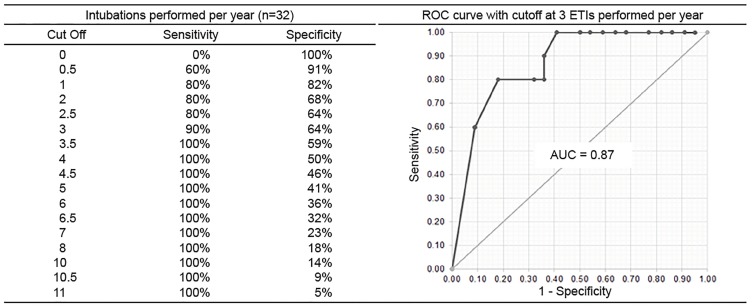
Sensitivity and specificity for various cut points represented as the number of endotracheal intubations performed annually. *ROC*, receiver operator characteristic; *ETI,* endotracheal intubation; *AUC,* area under the curve.

**Figure 2:**
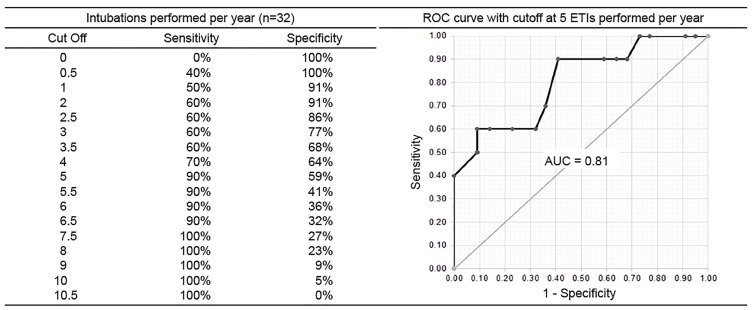
Sensitivity and specificity for various cut points represented as the number of ETIs supervised annually and the ROC curve for a cut point of five intubations supervised/year. *ROC*, receiver operator characteristic; *ETI,* endotracheal intubation; *AUC,* area under the curve.

ROC analysis identified, with good accuracy, that physicians would score at or above the proficiency cut score if they performed an average of at least three intubations annually (sensitivity = 90%, specificity = 64%, area under the curve [AUC] = .87, p = .001) or supervise an average of at least five intubations annually (sensitivity = 90%, specificity = 59%, AUC = .81, p = .006) over a period of two years (Figures 1 and 2).

## DISCUSSION

It is the public trust that gives physicians their status as professionals. When polled, 95% of respondents rated MOC for physicians as “important,” with a majority stating that regular testing to assess physician medical knowledge and periodically testing clinical performance and quality of care as being “very important.”^20^ Leach described skill acquisition and competence as a process, not a destination, with professional development needing to be a lifelong habit.^21^ This is because skill decay (the loss or degradation of acquired skills after periods of non-use) is a well-known phenomenon.

We ultimately identified two factors that correlated well with ETI performance–the number of intubations performed and the number of intubations supervised (on average per year for both). Specifically, physicians were at risk to fall below proficiency if they performed fewer than three or supervised fewer than five intubations per year on average. The ROC analysis allowed us to establish an optimal cut point for intubation frequency to predict proficient performance on the assessment. We chose cut points with higher sensitivity to avoid misclassification of “true positives,” ie, those who actually scored below the proficiency cut score on the assessment. We were unable to parse out the relative importance of performing vs supervising intubations as these metrics were exceedingly interconnected. It is unclear exactly how supervising intubations contributes to maintaining proficiency in the actual performance of ETI. However, neuroscience research on mirror neurons does suggest a physiologic basis for this phenomenon.^22^,^23^

Several studies have shown decay of critical cognitive and psychomotor skills in managing cardiopulmonary arrest.^24^,^25^,^26^ Major factors that influence the rate of decay are length of retention interval; degree of overlearning; task characteristics (closed loop vs open loop, cognitive vs physical, speed vs accuracy); methods of testing for original learning and retention; conditions of retrieval; instructional strategies or training methods; and individual differences in abilities.^27^ Historically, ETI was taught in the same place it needed to be performed – on patients in the clinical setting. While this method may positively influence some of the listed factors (original learning methods and conditions of retrieval), it is unlikely to provide the kind of experience that will lead to overlearning.

Ericsson et al. demonstrated that deliberate practice (rigorous practice with assessment and feedback) is the method of choice to gain expertise and avoid decay of a skill.^28^ For ETI, this would be most easily accomplished and assessed with simulation. Simulation-based assessments are increasingly integrated into medical education and have been proposed as the modality of choice to develop and assess procedural skill acquisition.^29^ Our study demonstrates replicable methodology using an airway simulator to assess performance. Obviously, simulation is not “real life”; however, it is the ethical alternative in which patient safety is not at risk and where confounding variables may be tightly controlled. Additionally, a simulation-based assessment carries greater face validity than the current practice of no assessment at all for this procedure. That said, the strong correlation between assessment scores and intubation experience suggests further construct validity of the assessment platform used in this study.

Similar to other sites,^30^ the PEM physicians in our cohort averaged less than one intubation per year, which is well below the threshold identified in our study. Not surprisingly, a prior survey of PEM directors revealed that 62% felt the number of ETIs performed were inadequate to maintain competency, and nearly half (48%) of the respondents reported that they use simulation to remediate or maintain competency.^31^ Ultimately, we chose not to exclude the PEM attendings, just as we chose not to exclude other cohorts that intubate less frequently (eg, physician administrators, researchers, or those working predominantly in less-acute zones), since the population of providers that infrequently perform ETI was specifically the group we were most concerned with regarding potential skill decay.

Board-certified EM and PEM physicians are expected to be able to perform airway management in adult and pediatric patients with requisite skill. Furthermore, the ACGME mandates the development of such skill as part of program requirements. Thus, we chose to include all providers who might be expected to perform an intubation on a patient with an adult-sized airway. Most dedicated pediatric emergency physicians treat patients with an upper age range from 18–25. We felt it would be inconsistent with the public trust placed in EDs for us to remove PEM providers from our cohort because they less frequently perform intubations. There is no published data showing PEM attendings have explicitly been assessed for procedural skill, and no data comparing their skill to general EM attendings. That said, in our cohort EM and PEM providers performed at both ends of the spectrum with a similar distribution of borderline performers to the overall cohort. Furthermore, in support of competency-based education, the expectation of educators is to train to a set standard regardless of subspecialty.^32^

Procedural re-credentialing is essentially automatic in our specialty, typically in two-year intervals, which is why we chose a two-year interval to analyze. Given the high-stakes nature of ETI, the results of this study may be used to help identify physicians who may benefit from refresher training in conjunction with re-credentialing. In our department, attendings who performed poorly were required to complete an airway refresher course at the department’s expense as well as local, simulation-based training. This approach was well received, and when re-assessed their scores dramatically improved.

It is well accepted that psychomotor skill acquisition and maintenance requires repetition. The surgical literature demonstrates this principle. Patient outcomes after surgical procedures have a clear association with the number of times that the surgeon has performed the procedure.^33^–^35^ Even when attempts to control for other factors have been considered, the number of times that a surgeon has performed a procedure remains strongly correlated to outcomes. This stands to reason: practice makes perfect.

Experience in years alone, however, does not predict a higher level of functioning. Our study showed a weak negative correlation between years of experience and assessed skill. Multiple previous studies have also shown that provider experience has an inverse relationship to many measures of clinical performance,^36^ and specifically in complex airway management.^37^ This implies that skills must be practiced with some minimal frequency. We cannot ethically dictate how many of our patients will need ETI, and so alternative methods of experience must be sought. Computer screen-based simulation may be an acceptable method for teaching some skills, but high-fidelity simulation has shown to assist in the retention of complex airway skills for up to one year.^38^

It is possible that the level of skill demonstrated by physicians on the airway simulator used in this study does not translate to a similar level of competence in the clinical arena. The use of simulation requires a “suspension of disbelief,” and there has been some concern raised that task trainers do not accurately replicate human anatomy.^39^–^41^ Using simulation for the assessment of competence needs to be authentic if it is to imply that the practitioner would perform similarly with real patients.^42^ However, research demonstrates that assessment in simulated environments can be reliable and valid.^43^,^44^ Specifically in airway management, studies have shown that assessment of competence corresponds to operational performance in the clinical setting.^45^,^46^ In addition, there is evidence supporting the use of mannequins for training, assessment, and maintaining competency.^47^,^48^

While faculty development may be ubiquitous in training institutions, generally it is focused on the domains relevant to career advancement such as teaching, administration, and research. The focus of developing more generalized knowledge, skills, and attitudes is limited to resident trainees. In our department, this initiative led to the formalization of an ongoing, robust, simulation-based faculty skills advancement curriculum that encompasses procedural (both novel and established), clinical, and cognitive skills. This has been well received by our faculty,^49^,^50^ and we hope this skills advancement curriculum will serve as a model for other organizations.

## LIMITATIONS

We abstracted intubation data from the electronic health record, making it possible that uncharted intubations may have been missed. Assessors and participants were both employed by the same department. This meant that although the assessors had no prior access to each provider’s intubation record, absolute blinding was impossible. It is unknown if this contributed to unconscious bias. Additionally, for ethical reasons assessments were conducted on an airway simulator as opposed to live patients. However, the strong correlation observed between physicians’ assessment scores and their average numbers of annual intubations suggests construct validity for this assessment, and internal consistency for the tool was very good.

Although general EM and PEM providers are expected to be able to intubate both adult and pediatric patients, we only tested providers on the adult-equivalent manikin. This study was performed at a single center with a small sample size and may reflect factors not found at other institutions. Lastly, the study was conducted at an academic ED, where the majority of intubations are supervised rather than performed by attending physicians. As such, there was significant variance among physicians with regard to the time between last performing an intubation and taking the assessment. This likely relates to why the assessment scores correlated particularly poorly with the time interval since last performing an intubation at our institution.

## CONCLUSION

Performing at least three or supervising at least five ETIs per year correlated with proficient performance on a skills assessment in our cohort. Our methodology is easily replicable and can be extrapolated across a wide range of procedures in future studies. Since simulation training has become widely available, we advocate for this modality as a platform for active maintenance and advancement of procedural skills. This approach was well received in our department.

## Figures and Tables

**Table 1:** 11-item intubation checklist.

Assembly of equipment: □ Suction □ Correct-sized endotracheal tube and blade □ Back-up tube and blade □ Rescue device □ Stylet □ Confirmation device (EDD, EtCO_2_ detector, etc.) Discrete actions: □ Evaluates airway anatomy and mobility □ Positions appropriately □ Articulates RSI meds □ Does not rock laryngoscope handle backwards on insertion □ Inserts tube to correct depth

*EDD*, esophageal detector device; *EtCO_2_,* end-tidal carbon dioxide; *RSI,* rapid sequence intubation.

**Table 2:** Summary of practice setting and provider characteristics.

Practice setting	Faculty specialty	Physician	Total supervised	Total performed	Years post-residency
Academic urban	General EM	1	11	14	4.5
		2	16	16	4.5
		3	18	20	3.5
		4	6	10	0.5
		5	7	6	8.5
		6	10	12	8.5
		7	8	0	8.5
		8	10	2	10.5
		9	16	21	3.5
		10	5	0	30.5
		11	5	9	0.5
		12	10	13	16.5
		13	6	1	12.5
		14	16	7	5.5
		15	20	23	4.5
		16	10	8	2.5
		17	4	6	2.5
		18	7	2	6.5
		19	8	1	9.5
		20	15	7	7.5
		21	8	1	15.5
		22	13	5	3.5
		23	13	13	2.5
		24	12	4	4.5
	Pediatric EM	25	1	0	3.5
		26	1	0	17.5
		27	0	0	12.5
		28	1	2	3.5
		29	0	0	33.5
		30	2	0	3.5
		31	0	1	7.5
		32	0	0	10.5

	Total precepted	Total performed	Years post-residency
General EM mean (n=24)	10.6	8.4	7.4
Pediatric EM mean (n=8)	0.6	0.4	11.5
Total mean (n=32)	8.1	6.0	8.4

*EM*, emergency medicine.

**Table 3:** Comparison of emergency medicine and pediatric emergency medicine providers’ assessment scores in intubation skills.

ETI Assessment Score	Mean	Median	IQR	Standard Deviation	Range (min)	Range (max)
All EM Attendings (n=44)	81%	86%	76–91%	16%	33%	100%
Adult EM Attendings (n=33)	85%	86%	81–95%	14%	33%	100%
PEM Attendings (n=11)	69%	76%	60–79%	17%	33%	86%

*ETI*, endotracheal intubation;* IQR,* interquartile range; *EM,* emergency medicine; *PEM*, pediatric emergency medicine.
